# Learning Curve in Endoscopic Pituitary Surgery: Is Progress over Time Always Guaranteed? A Consecutive Series of 123 Cases from a Single Center

**DOI:** 10.3390/jcm15020569

**Published:** 2026-01-10

**Authors:** Marta Koźba-Gosztyła, Anastasija Krzemińska, Tomasz Szczepański, Bogdan Czapiga

**Affiliations:** 1 Department of Neurosurgery, Wroclaw 4th Military Clinical Hospital, 50-981 Wroclaw, Poland; martakozba1@o2.pl (M.K.-G.); tomek-md@wp.pl (T.S.); bogdan.czapiga@pwr.edu.pl (B.C.); 2 Faculty of Medicine, Wroclaw University of Science and Technology, Grunwaldzki Square 11, 51-377 Wrocław, Poland

**Keywords:** learning curve, pituitary surgery, macroadenoma, transsphenoidal, complications

## Abstract

**Objectives:** To characterize the learning curve of endoscopic transsphenoidal pituitary adenoma surgery performed by a single neurosurgeon, assess how operative time, resection rates, and clinical outcomes evolved with experience, and identify tumor-related factors influencing surgical performance. **Methods:** This retrospective study included 123 consecutive endoscopic transsphenoidal pituitary adenoma resections performed between 2018 and 2025. Cases were divided into quartiles according to chronological order. Clinical, radiological, endocrinological, and operative variables were analyzed. Gross total resection (GTR), biochemical remission, postoperative complications, and visual and cranial nerve outcomes were compared between quartiles. A segmented linear regression model was applied to identify changepoints in the operative-time learning curve. Statistical significance was set at *p* < 0.05. **Results:** The mean operative time decreased by 31.8%, from 160.8 min in Quartile 1 to 109.7 min in Quartile 4. Segmented regression revealed two changepoints at cases 47 and 85, defining three learning phases: a steep improvement phase, a consolidation phase, and a plateau. GTR was achieved in 51.2% of patients and did not significantly differ across quartiles. For Knosp 0–2 tumors, GTR was 76.1% overall; for Knosp 3–4 tumors, 30%. Tumor diameter, Knosp grade, and sphenoid sinus invasion were strongly associated with lower GTR rates (all *p* < 0.05). Biochemical remission was achieved in 74.2% of patients with functional adenomas. New or worsened postoperative pituitary insufficiency significantly decreased across quartiles (*p* < 0.001). Rates of postoperative diabetes insipidus (30.8%) and CSF leak (6.5%) were comparable with published literature and showed no consistent temporal trend. **Conclusions:** A clear learning curve exists in endoscopic pituitary surgery, with operative proficiency achieved after approximately 50 cases and an experienced plateau after ~90 cases. Surgical experience significantly reduced operative time and postoperative pituitary insufficiency but did not influence GTR rates, likely due to a high and increasing proportion of large tumors with cavernous sinus invasion. Tumor size, Knosp grade, and sphenoid sinus invasion were identified as major determinants of surgical complexity and should be accounted for when evaluating learning curves and surgical outcomes.

## 1. Introduction

Endoscopic surgery has revolutionized the management of pituitary tumors over the past few decades. The concept of the transsphenoidal route to reach the sella has existed for nearly a century. Although Harvey Cushing and Oskar Hirsch built on the work of their predecessors, their transsphenoidal procedures became the two most popular techniques and laid the foundation for modern transsphenoidal surgery [1]. The introduction of the microscope to these techniques by Jules Hardy in 1962 improved the magnification and illumination of the operative field [2]. The operative microscope soon became the standard for transsphenoidal pituitary tumor resections until the benefits of using an endoscope were discovered in 1992 [3,4]. Nowadays, the literature comparing endoscopic and microscopic pituitary surgery favors the endoscopic approach. Although major outcome measures (extent of tumor resection, changes in hormone levels) do not differ between the two approaches, according to some authors, complications, time in the operating room and hospital stay, and patient discomfort are significantly less with the endoscopic approach [5,6].

However, as with any technically demanding procedure, mastering endoscopic pituitary surgery requires a significant learning curve. Understanding this learning process is essential—not only for ensuring patient safety and achieving optimal surgical results, but also for designing effective training programs and assessing surgical competency. Over the past 30 years, multiple authors have reported techniques, complications, and outcomes of fully endoscopic pituitary surgery, but most of their articles were not related to assessing the learning curve [7].

The objectives of this study were to characterize the learning curve of endoscopic transsphenoidal pituitary adenoma surgery and to identify the factors influencing surgical performance during its progression, while acknowledging that outcomes such as gross total resection (GTR) rate and complications may be strongly confounded by tumor complexity.

Specifically, we aimed to (1) analyze how operative time, extent of tumor resection, and outcomes evolved with experience, (2) assess the relationship between tumor characteristics and surgical outcomes, and (3) determine the number of procedures required to achieve technical proficiency.

By examining a consecutive single-surgeon series of 123 operations, this study seeks to provide a comprehensive evaluation of both the technical and clinical dimensions of the learning process in endoscopic pituitary surgery.

## 2. Materials and Methods

### 2.1. Patient Population

This retrospective study included 123 consecutive transsphenoidal endoscopic pituitary adenoma resections performed at a large tertiary medical center between January 2018 and August 2025. Only cases with a final histopathological diagnosis of pituitary adenoma were included; tumor biopsies and non-adenomatous lesions were excluded.

Data were collected from electronic medical records and included demographic information, prior treatment history, radiological and intraoperative findings, and postoperative outcomes.

All patients had a minimum follow-up period of three months.

For the purpose of analysis, all cases were divided into quartiles according to the chronological order of surgery.

### 2.2. Surgical Technique

All operations were performed by a single neurosurgeon: the first author (123 surgeries), with the first 23 procedures under the supervision of the senior author. Both surgeons had prior experience in neurosurgery. The senior author had performed microscopic transsphenoidal pituitary surgeries for 14 years (approximately 150 surgeries) before adopting the endoscopic technique.

No otolaryngologist participation was involved in any of the analyzed cases.

MRI-based neuronavigation was employed only in patients who had undergone previous surgery via the same transsphenoidal route and in microadenomas.

**Figure 1:**
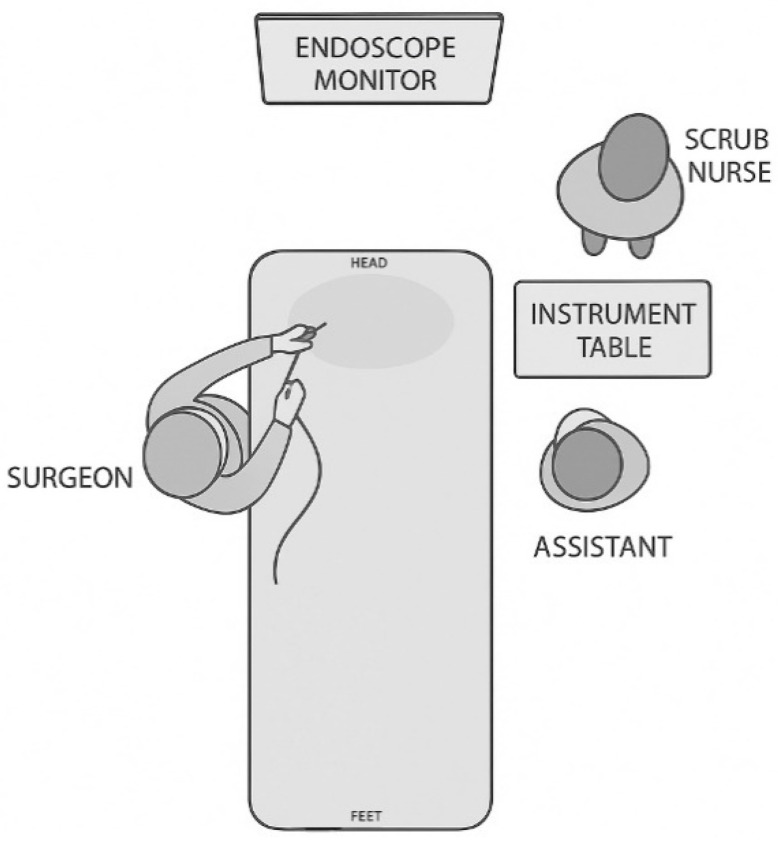
The operative setup during endoscopic transsphenoidal pituitary surgery.

The operative setup is illustrated in Figure 1.

The procedure is performed with the patient in the supine position, with the head elevated at approximately 45°. The nasal cavities are irrigated with povidone-iodine solution. Cotton pledgets soaked in adrenaline solution are placed in both nasal cavities to achieve mucosal decongestion. After removal of the pledgets, a 0° rigid endoscope is introduced to explore the single nasal passage. The middle turbinate is gently shifted, and the natural sphenoid ostium is identified. The right nostril was preferred in patients without nasal septum deviation. In cases with septal deviation, the side for the surgical approach was selected according to the direction of the deviation to ensure a wider working corridor.

The mucosa around the sphenoid ostium is coagulated using monopolar cautery, including the posterior part of the nasal septum inferior to the olfactory epithelium. With a diamond drill, the anterior wall of the sphenoid sinus is widened, and the nasal septum is detached from the sphenoid rostrum and mobilized to the left side.

The endoscope is then advanced into the sphenoid sinus. The inter-sinus septum is removed, and the sinus mucosa is partially resected to expose the sellar floor. The thin bony sellar floor is opened using a diamond drill and enlarged with Kerrison rongeurs. The dura mater is incised, leading in most cases to spontaneous partial tumor evacuation. The adenoma is removed using curettes and pituitary rongeurs.

Subsequently, a 30° endoscope is introduced to inspect the cavernous sinus regions bilaterally. Hemostasis is achieved using Spongostan (Ethicon Inc., Somerville, USA). A small fragment of abdominal fat is taken from the left periumbilical region and, together with fibrin glue, used for sellar reconstruction. An additional layer of abdominal fat is then placed and covered with fibrin glue to achieve a multilayer reconstruction. Finally, nasal packing is placed bilaterally in both nasal cavities.

The core surgical technique, endoscopic approach, and reconstruction strategy (fat-glue multilayer closure) remained consistent throughout the study period; no major changes in instrumentation or reconstruction philosophy were introduced.

### 2.3. Collected Variables

Extracted data were entered into a spreadsheet using Microsoft Excel (2019; Microsoft Corporation, Redmond, WA, USA). The following variables were collected and analyzed:

- Demographic data: sex and age.
- Comorbidities: presence of diabetes mellitus and smoking status.
- Preoperative symptoms: headache, cerebrospinal fluid (CSF) leak, visual disturbances, pituitary insufficiency, and cranial nerve palsy related to the cavernous sinus (CS CN).
- Radiological findings (MRI): tumor size, Knosp grade, and sphenoidal sinus invasion.
- Hormonal status of the tumor: prolactinoma, ACTH-secreting, GH-secreting, or non-functioning adenoma.
- Postoperative neurological and endocrine outcomes: visual disturbances, CS CN palsy, pituitary insufficiency, biochemical remission (in patients with functioning adenomas).
- Postoperative complications: diabetes insipidus, CSF leak, infection.
- Operative parameters: date of surgery, operative time, length of hospital stay (LHS), extent of resection, tumor recurrence and duration of follow-up.

Pre- and postoperative MRI scans (performed up to 9 months postoperatively) were reviewed by the authors.

Intraoperative CSF leakage was not systematically recorded during the early study period and was therefore not included in the analysis.

The maximum tumor diameter in any plane was recorded to represent tumor size. Microadenoma was defined as a lesion with a maximum diameter ≤ 10 mm, whereas macroadenoma was defined as a tumor exceeding 10 mm.

GTR was defined as the absence of a visible tumor on postoperative MRI, assessed on both contrast-enhanced T1-weighted and T2-weighted sequences. Subtotal resection (STR) was defined as the presence of residual tumor on postoperative MRI.

Sphenoid sinus invasion was defined as radiological evidence of tumor extension through the sellar floor into the sphenoid sinus, involving ≥50% of the sinus cavity on preoperative MRI.

Biochemical remission was defined as follows:

- for prolactinomas, normalization of serum prolactin (PRL) levels to <15.2 μg/L;
- for GH-secreting adenomas, normalization of serum growth hormone (GH) levels to <1 μg/L or insulin-like growth factor 1 (IGF-1) to <186 μg/L;
- for ACTH-secreting adenomas, normalization of morning serum cortisol to <507 nmol/L, measured 3–7 days postoperatively.

Tumor recurrence was defined as the appearance of new tumor growth after GTR, confirmed on follow-up MRI. In patients with residual tumor (STR), any increase in tumor volume on follow-up imaging was classified as tumor progression.

Postoperative complications were assessed both during hospitalization and at long-term follow-up. A postoperative CSF leak was defined as the presence of clear CSF rhinorrhea confirmed by a positive β-trace protein test, requiring reoperation for surgical repair.

### 2.4. Statistical Analysis

All statistical analyses were performed using Statistica 14.2.0 (TIBCO Software Inc.). Continuous variables were expressed as mean ± standard deviation (SD) and compared between quartiles using the one-way analysis of variance (ANOVA) or Kruskal–Wallis test when the assumption of normality was not met.

Categorical variables were presented as counts and percentages and compared using the Pearson χ^2^ test or Fisher’s exact test, as appropriate. The nonparametric Mann–Whitney U test was applied to evaluate associations between tumor characteristics and surgical outcomes. The *p*-value < 0.05 was considered statistically significant.

To evaluate the learning curve, consecutive surgical cases were divided into quartiles according to chronological order. Additionally, a segmented (piecewise) linear regression analysis was applied to model the relationship between consecutive operative cases and operative duration. All possible pairs of breakpoints across the ordered series were iteratively tested, and the model with the lowest residual sum of squares (RSS) was selected as the optimal fit. This approach allowed the identification of one or more changepoints (phases) in the learning curve that corresponded to major improvements in operative efficiency.

Missing data were excluded from statistical comparisons on a per-variable basis (pairwise deletion). All statistical tests were two-tailed.

The study was conducted in accordance with the Ethical Principles for Medical Research Involving Human Subjects, as outlined in the Declaration of Helsinki (issued in 2004, revised in 2008 and 2013). The recommendations of the STROCSS (Strengthening the Reporting of Observational Cohort Studies in Surgery) statement were followed for reporting the results.

## 3. Results

### 3.1. Patient Demographics and Clinical Characteristics

A total of 123 patients who underwent endoscopic transsphenoidal pituitary adenoma resection were included in the analysis. There were 69 men (56.1%) and 54 women (43.9%), with a mean age of 61.3 ± 13.9 years. To assess the learning curve, patients were divided into quartiles according to the chronological order of surgery, with a relatively even distribution across groups (Q1 = 31 [25.2%] (years 2018–2019, Q2 = 28 [22.8%] (years 2020–2021), Q3 = 32 [26.0%] (years 2022–2023), Q4 = 32 [26.0%] (years 2024–2025)). There were no differences in age or sex between quartiles (see Table 1).

Nine patients (7.3%) underwent previous surgery for pituitary adenoma. Previous surgeries had been performed at other centers via transsphenoidal approach using either endoscopic or microscopic techniques.

Two patients (1.6%) had a history of radiotherapy to the pituitary region before the index procedure. Among the study cohort, 26 patients (21.1%) were diagnosed with diabetes mellitus, and 39 (31.7%) were active smokers.

The most common preoperative symptom was subjective visual deterioration, reported by 64 patients (52.0%), followed by headache in 44 patients (35.8%).

A preoperative cerebrospinal fluid (CSF) leak was identified in one patient (0.8%).

The mean length of hospital stay ranged from 6.0 to 9.5 days, with no clear trend across quartiles.

### 3.2. Tumor Characteristics

The mean maximum tumor size was 29.4 mm, ranging from 28.3 to 30.6 mm (*p* = 0.84) between the quartiles.

Among the 123 pituitary adenomas, the majority were non-functioning pituitary adenomas (NFPA) (*n* = 89, 72.4%). Prolactinomas accounted for 21 cases, GH-secreting adenomas for 13 cases, and ACTH-secreting adenomas were rare, with only 1 case identified. One patient had an adenoma that was secreting both prolactin and growth hormone. There were 117 (95.1%) macroadenomas.

Cavernous sinus invasion (Knosp 3 and 4) was present in 51.6%, 57.2%, 56.2%, 62.5% in the first through last quartiles, respectively.

Sphenoid sinus invasion was present in 53 (43.1%) cases.

**Figure 2:**
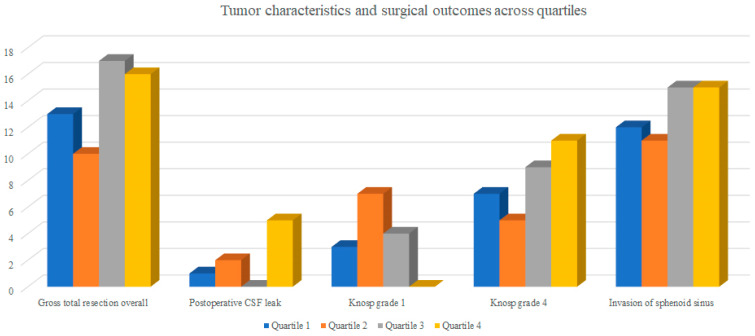
Tumor characteristics and surgical outcomes across quartiles. (Bar chart illustrating selected tumor characteristics and key surgical outcomes in four quartiles of surgical experience. With increasing experience, the proportion of less invasive tumors (Knosp grade 1) decreased, while the proportion of highly invasive lesions (Knosp grade 4, invasion of the sphenoid sinus) increased, indicating a gradual shift toward surgically more challenging cases.

There were no statistically significant differences in tumor size, Knosp grade, or sphenoid sinus invasion between quartiles (all *p* > 0.05) as presented in Table 2 and Figure 2. However, certain trends were observed.

The rate of postoperative cerebrospinal fluid (CSF) leak showed an increasing trend (*p* = 0.067).

Gross total resection (GTR) remained high across all quartiles, with a mild decrease observed in later cases.

Overall, the data suggest that as surgical experience advanced, case complexity increased while maintaining stable resection outcomes.

Low-invasiveness lesions (Knosp 1) were more frequent in the early phase of the learning curve and became rare in later quartiles (9.7%, 25.0%, 12.5%, and 0% for Q1–Q4, respectively; *p* = 0.053).

Conversely, the proportion of highly invasive tumors (Knosp 4) showed a gradual increase across quartiles (22.6%, 17.9%, 28.1%, and 34.4%), although this trend was not statistically significant (*p* = 0.67).

### 3.3. Operative Time

The mean operative time was 126.9 ± 43.8 min. Operative time decreased from 160.8 ± 54.5 min in the first quartile to 109.7 ± 24.1 min in the fourth quartile, representing an overall 31.8% reduction in operative duration.

**Figure 3:**
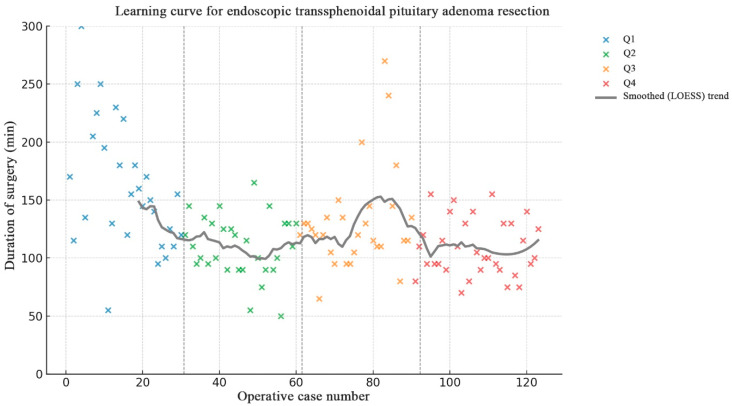
Learning curve for endoscopic transsphenoidal pituitary adenoma resection. (Scatterplot demonstrating the duration of consecutive surgeries (1–123) performed via the endoscopic transsphenoidal approach. Each color represents one quartile of surgical experience (Q1–Q4). The gray curve shows the smoothed (LOESS) trend. A clear decrease in operative time is observed over the course of the series, suggesting progressive improvement in surgical efficiency.

A significant reduction in operative time was observed after the first 30–35 cases, followed by a plateau phase with stable mean duration around 110–130 min (*p* < 0.001 between Q1 and Q2; nonsignificant thereafter) (Figure 3).

**Figure 4:**
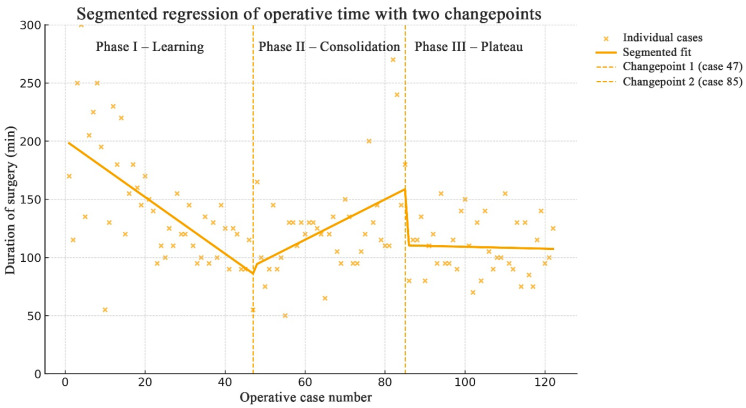
Segmented regression analysis of operative time learning curve with two changepoints. The segmented regression model of operative time for 123 endoscopic transsphenoidal pituitary adenoma resections demonstrated two changepoints, at cases 47 and 85, dividing the learning curve into three distinct phases; A marked reduction in operative time occurred during the initial phase (up to case 47), followed by a gradual consolidation phase (cases 48–85) and a plateau phase thereafter, with stable mean duration around 110–130 min; each dot represents a single operation, and the solid line shows the fitted piecewise linear trend. Dashed vertical lines indicate changepoints.

Learning curve analysis was performed using segmented (piecewise) linear regression to model the relationship between consecutive operative cases and operative duration. The model iteratively tested all possible pairs of changepoints (breakpoints) across the ordered series of 123 cases, fitting separate linear regressions for each segment. The combination of changepoints that minimized the residual sum of squares (RSS) was selected as the optimal model. Two changepoints were identified at cases 47 and 85, dividing the learning curve into three phases (Figure 4). A marked reduction in operative time occurred up to case 47 (representing the steepest segment of the curve), followed by a consolidation phase until case 85 and a subsequent plateau thereafter.

### 3.4. Comparison of Operative Times Between Functioning and Non-Functioning Adenomas

When comparing hormonally active and non-functioning pituitary adenomas, both groups demonstrated a significant reduction in operative time across quartiles, although the pattern of improvement differed.

For non-functioning adenomas, operative duration significantly declined across the series (F(3,85) = 6.61, *p* = 0.00045). The mean operative time decreased from 157.8 min in Quartile 1 to 112.0 min in Quartile 2, with stabilization around 110–126 min in later quartiles.

For functioning adenomas, the trend was also significant (F(3,30) = 4.33, *p* = 0.012), but the pattern was less linear. Operative time sharply decreased from 163.3 min in Quartile 1 to 102.5 min in Quartile 2, then increased in Quartile 3 (134.4 min), before decreasing again in Quartile 4 (107.7 min).

Between-group comparison:

- In Quartile 1, operative times were comparable between functioning (163.3 min) and non-functioning adenomas (157.8 min).
- In Quartile 2, both groups showed marked reductions, with very similar mean durations (102.5 min vs. 112.0 min, respectively).
- In Quartile 3, functioning adenomas required clearly longer operations (134.4 min) than non-functioning adenomas (126.3 min).
- In Quartile 4, operative times converged again (107.7 vs. 110.7 min).

### 3.5. GTR Rate

For the entire cohort, GTR was achieved in 63 (51.2%). For the Knosp 0–2 group, it was 76.1%. For the Knosp 3–4 group, it was 30%.

The overall rate of GTR did not differ significantly between the four quartiles (48.1%, 38.5%, 53.1%, and 51.6% for Q1–Q4, respectively; *p* = 0.72). Although minor fluctuations were observed, no clear trend toward improvement or deterioration in GTR rates across consecutive quartiles was detected.

When tumors were stratified according to Knosp classification, similarly stable results were obtained within each subgroup. For Knosp 0–2 adenomas, GTR was consistently high across quartiles (81.8%, 60.0%, 78.6%, and 81.8% for Q1–Q4, respectively), with no statistically significant differences (*p* = 0.69). For Knosp 3–4 adenomas, GTR remained lower but relatively comparable across quartiles (25.0%, 25.0%, 33.3%, and 35.0% for Q1–Q4), also demonstrating no significant temporal variation (*p* = 0.86).

Tumor recurrence during the follow-up period was rare, occurring in 2.46% of cases (*p* = 0.24).

### 3.6. Endocrinological Outcomes

Biochemical remission (BR) was achieved in 74.2% (26/35) of patients with functional adenomas, ranging from 50% to 100% across quartiles (*p* = 0.37) (Table 2). Among GH-secreting adenomas, BR was achieved in 84.0% (11/13) of patients, while 71% (15/21) of patients with PRL-secreting adenomas reached BR. BR was not achieved in the single patient with an ACTH-secreting adenoma.

**Figure 5:**
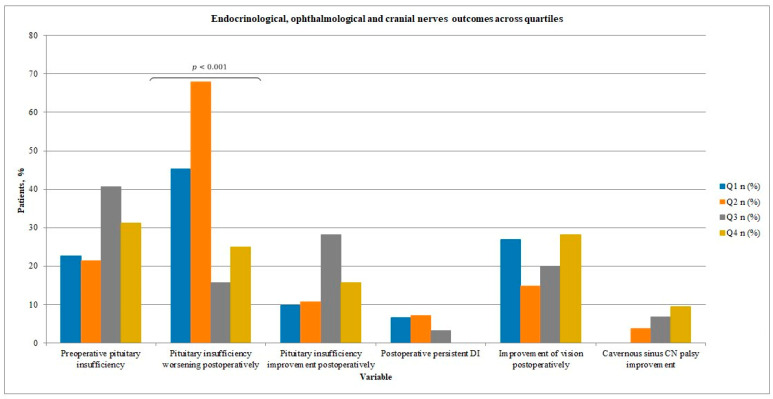
Endocrinological, ophthalmological and cranial nerves outcomes across quartiles. (Comparison of endocrinological outcomes between quartiles of consecutive endoscopic transsphenoidal pituitary adenoma resections; The rate of preoperative pituitary insufficiency was comparable among groups; A significantly higher proportion of postoperative pituitary function worsening was observed in the early phase of the learning curve (particularly in quartile 2; *p* < 0.001), followed by a marked decrease in subsequent quartiles; Improvement of pituitary function and occurrence of persistent diabetes insipidus showed no significant differences between groups; The proportion of patients with preoperative or postoperative visual disturbances gradually decreased over time, although without reaching statistical significance (*p* > 0.05).

Rates of preoperative pituitary insufficiency ranged from 21.4% to 40.6% across quartiles (*p* = 0.31) (see Table 3 and Figure 5). Overall, new or worsened hormonal dysfunction was observed in 37% of cases in our study group, while hormonal status improved in 16% of patients. Notably, the rate of new or worsened pituitary insufficiency postoperatively differed significantly between quartiles, showing a tendency to decrease (45.2%, 67.9%, 15.6%, and 25.0% for Q1–Q4; *p* < 0.001).

### 3.7. Vision and Cranial Neuropathy Outcomes

There were available short-term post-operative visual outcomes (up to 1 month, performed during a hospital stay). Long-term visual outcomes (up to one year) were available only in 57 patients, so the statistical analysis was performed for short-term outcomes.

Pre-operative visual impairment was present in 59.3% of patients, whereas pre-operative cranial neuropathy was uncommon, occurring in only 12.2% of cases.

Vision and cranial neuropathy outcomes across quartiles are summarized in Table 3 and visualized in Figure 5. The proportion of patients with preoperative or short-term postoperative visual disturbances gradually decreased over time, although without reaching statistical significance (*p* > 0.05). In the last period, 53% of patients with preoperative visual deficits improved in the short post-operative term. A single case of new postoperative visual deficit occurred in Q4.

The incidence of cranial neuropathy also showed a downward trend, with no significant intergroup differences.

### 3.8. Complication Rate

Postoperative diabetes insipidus (DI) was predominantly transient and occurred in 30.8% of patients (19.4–39.3% across quartiles). Persistent DI was uncommon, occurring in 4% of patients (0–7.1% across quartiles; *p* = 0.46). No clear trend was observed in the incidence of either transient or permanent postoperative DI.

We did not collect information on intraoperative CSF leakage because it was not always available in the medical records; however, such leakage was not uncommon. As a rule, we did not use lumbar drainage when an intraoperative CSF leak occurred. In all cases, a fat–glue multilayer reconstruction was performed. Postoperatively, CSF leakage occurred in 8 patients (6.5%), with 1, 2, 0, and 5 cases in the respective quartiles, demonstrating a non-significant increasing trend. All affected patients required reoperation. Fat–glue multilayer reconstruction was again performed, without the use of lumbar drainage.

There were 3 (2.4%) patients with meningitis secondary to CSF rinorrhea: one in Quartile 2 and two in Quartile 4.

### 3.9. Factors Influencing Surgical and Functional Outcomes

The presence of sphenoid sinus invasion was significantly associated with a lower rate of gross total resection (U = 1264.0, Z = 2.10, *p* = 0.036; Mann–Whitney U test).

Knosp grade differed significantly between patients who achieved gross total resection and those with subtotal resection. Patients with GTR had significantly lower Knosp grades compared with the STR group (U = 173.0, Z = 4.20, *p* < 0.001). This indicates that cavernous sinus invasion severity is a strong negative predictor of complete resection.

Maximum tumor size differed significantly between patients with gross total resection and those with subtotal resection. Tumors in the STR group were substantially larger, with a very strong statistical significance (U = 494.5, Z = 6.78, *p* < 0.001). This confirms tumor size as an important limiting factor for achieving complete resection.

## 4. Discussion

The concept of the surgical learning curve represents a fundamental framework for evaluating the acquisition of technical proficiency and the optimization of outcomes in each branch of surgery, including endoscopic transsphenoidal surgery (ETS). For a neurosurgeon accustomed to the microscopic technique, which provides a three-dimensional view and enables operating with a two-handed technique, this minimally invasive approach brings certain challenges, such as a two-dimensional view, the limitation of using one hand to hold the endoscope, the periodic fogging of the endoscope lens by blood, and difficulties in controlling significant hemorrhage.

The learning curve in this context delineates the relationship between the surgeon’s cumulative experience—expressed by the number of procedures performed—and objective measures of performance, such as operative time, extent of resection, intraoperative complications, CSF leak rates, and postoperative outcomes.

Although more than three decades have passed since the introduction of endoscopy in pituitary tumor surgery, there are still relatively few studies that describe the learning curve in this field. In a review by Candy et al. (2023), the authors identified only ten studies that adequately evaluated how outcomes in endoscopic pituitary surgery change as surgeons gain more experience [7].

Typically, the learning curve exhibits a sigmoidal pattern, beginning with an initial phase of technical adaptation and longer operative times, followed by a period of rapid improvement in efficiency and safety, and eventually reaching a plateau reflecting procedural mastery and consistent results. According to some authors, the late plateau is not flat but shows a persistent positive slope, indicating continued improvement [8,9]. In this context, the end of the steep segment of the learning curve is of particular importance, as it signifies that the surgeon has achieved sufficient experience in endoscopic pituitary tumor surgery, while maintaining continuous professional development and refinement of surgical skills.

Our study included a series of 123 endoscopic transsphenoidal pituitary adenoma resections performed by a single neurosurgeon at a tertiary care center. The results demonstrate improved operative efficiency with increasing surgical experience, while maintaining stable surgical safety and endocrinological outcomes. Variables that showed a statistically significant reduction were operative time and the rate of de novo pituitary insufficiency. The proportion of patients with postoperative visual improvement and recovery from cranial nerve palsy gradually increased over time, although these changes did not reach statistical significance.

### 4.1. Duration of Surgery

The mean duration of surgery decreased from 160 min in the first quartile to approximately 110 min in later periods, representing an overall reduction of about 32% in operative time, which was statistically significant. A comparable downward trend in operative duration has also been reported in previous studies, ranging from 13% to 40% [10,11,12,13,14,15,16,17]. Additionally, Leach et al. reported that the duration of surgery for functioning adenomas was longer than for non-functioning adenomas [11]. In the study by Huang et al., operative time for the giant adenoma group and for the Knosp grade 3–4 adenoma group also decreased over time, although this reduction did not reach statistical significance, and the downward trend was relatively slower [10].

In our study, a marked decrease in operative duration was observed up to case 47, which constituted the steepest part of the learning curve, followed by a consolidation phase until case 85 and a subsequent plateau thereafter. The steepest segment of the curve indicates a rapid gain in surgical proficiency, while the plateau reflects the achievement of mastery. One of the key goals in studying surgical learning curves is to determine the minimum number of cases after which a surgeon can be considered proficient or, as a subsequent step, experienced [8]. In line with these findings, in our series, after approximately 50 endoscopic transsphenoidal surgeries, a surgeon can be regarded as well-trained or proficient, and after around 90 cases as experienced.

Some previous studies have estimated that the learning curve for achieving proficiency in endoscopic transsphenoidal surgery ranges between 17 and 34 cases [15,16]. Two studies published in 2008 and 2013 estimated that approximately 40 cases were required to achieve a significant reduction in operative time during ETS procedures [17,18]. In contrast, Shikary et al. reported that approximately 120 procedures were required for a surgical team to reach a plateau, with a mean operative time of around 125 minutes [19]. In the study by Leach et al., a decrease in operative duration was observed only for non-functioning pituitary adenomas and became apparent after approximately 50 cases had been performed [11]. In our study, operative times decreased significantly in both functioning and non-functioning adenomas; however, the improvement in functioning adenomas was less uniform, with a transient increase in the third quartile. Non-functioning adenomas showed a more consistent downward trend.

### 4.2. Rate of GTR

GTR rate in endoscopic surgical series varied from 59% to 92% [20,21,22,23]. In our series, GTR was achieved in 63 (51.2%) of cases. The overall rate of GTR did not differ significantly between the four quartiles (48.1%, 38.5%, 53.1%, and 51.6% for Q1–Q4, respectively). Additionally, there was no clear trend toward improvement or deterioration. Unlike our findings, many authors have reported a significant improvement in the extent of tumor resection over time, whereas others have demonstrated an improvement that did not reach statistical significance [8,10,11,12,17,24,25,26,27,28]. Kim at al. revealed that during the first period of their study, the GTR rate was only 63.0%, but after 100 cases of experience, the surgical outcomes improved to 80.1% [28]. In their study, however, the proportion of patients with cavernous sinus invasion (Knosp 3 and 4) was 43.5%. In the study by Younus et al., this proportion was 19%, 25%, 29%, and 27% across consecutive periods [8]. In other published series, the percentage of such cases also did not exceed 40% [10,25].

In our cohort, the stable GTR rate observed across quartiles should be interpreted in the context of progressively increasing tumor complexity, particularly the high and rising proportion of Knosp grade 3–4 adenomas in later periods. Cavernous sinus invasion was present in 51.6%, 57.2%, 56.2%, and 62.5% of cases, respectively, which was exceptionally high. The mean maximum tumor size was also relatively high, reaching 29.4 mm, whereas other studies have reported values of 23.3 mm or 27.5 mm [8,25]. Tumor size and cavernous sinus invasion were identified as significant determinants of resection completeness. Specifically, larger tumors and those invading the cavernous sinus were less likely to be completely resected, reflecting the well-known challenges associated with parasellar or extrasellar extension. In our study, tumor diameter, Knosp grade, and sphenoid sinus invasion were significantly associated with a lower rate of gross total resection. Kim at al showed a 2.29-fold higher risk for tumor control failure per 1 cm increase in tumor size, which was also similar to the Tabaee et al. study (3-fold increase for every 1 cm increase in tumor size) [28,29]. Dehdashti et al. showed that the degree of GTR for non-functioning pituitary adenomas (NFPAs) without cavernous sinus involvement was 97%, whereas the GTR rate for NFPAs with cavernous sinus involvement was 0% [21].

This lack of improvement in GTR across consecutive quartiles in our series is likely related to the exceptionally high proportion of large tumors with cavernous sinus invasion in our cohort, ranging from 51.6% to 62.5%, which is higher than in most published series (19–43.5%). This highlights that, while surgical experience is important, the learning curve for achieving complete resection may be masked in cohorts dominated by complex, high-risk tumors. The absence of an increasing GTR rate over time does not imply a lack of surgical progression but rather reflects a shift toward more complex cases.

### 4.3. Endocrine Outcome

According to the literature, new or worsened hormonal dysfunction after endoscopic transsphenoidal surgery (ETS) can occur in up to 58% of cases [28]. Therefore, the Endocrine Society guidelines recommend that hypopituitarism should be considered only a relative indication for surgery in patients with nonfunctioning pituitary adenomas (NFPAs) [30]. In our study, overall hormone status improved in 16% of patients and deteriorated in 37% after surgery. Slightly better outcomes were reported by Kim et al., who observed improvement in 21.7% and deterioration in 32.9% of patients [28].

Several studies are available that report on the rate of postoperative new or worsened pituitary insufficiency between early and late surgical groups. In the study by Leach et al., this rate increased from 17% to 25%, whereas Boetto et al. noted a decrease from 13% to 4.3% [11,25]. Younus et al. revealed that, for nonfunctioning adenomas, the rate of normal endocrine outcome increased across quartiles from 81% to 84% to 87% to 90%, a non-statistically significant trend [8]. In our study, the rate of new or worsened postoperative pituitary insufficiency differed significantly between quartiles, initially being high and showing a statistically significant tendency to decrease (45.2%, 67.9%, 15.6%, and 25.0% for Q1–Q4; *p* < 0.001), which may be explained by increasing surgical experience and the progression along the learning curve.

FPAs constitute a minority of tumors in studies evaluating the outcomes of endoscopic transsphenoidal surgery (ETS) [8,10]. Due to the relatively small number of cases in each hormonal subgroup, meaningful statistical analysis and reliable learning curve assessment are usually precluded. In a large study of 600 patients, the overall biochemical remission rate was high and increased significantly over time from 68% to 90% when all functional tumors were analyzed together [8]. In our cohort, biochemical remission was achieved in 74.2% of patients overall. The highest BR rate was observed in GH-secreting adenomas, reaching 84%. This finding represents a substantial improvement compared with an earlier study reporting an overall remission rate of only 18% in acromegaly patients [31]. Similar outcomes, with an overall remission rate of 82% in GH-secreting adenomas, were reported by Leach et al., who achieved better results than in prolactinomas, where the overall remission rate was only 56% [11]. This discrepancy is likely because the surgical goal for PRL-secreting adenomas is often maximal cytoreduction rather than aggressive GTR, which may affect the surgical cure rate. Therefore, the trend in BR rates for PRL-secreting adenomas is less suitable for assessing a surgeon’s operative skill [10].

### 4.4. VF Outcome

In our study, the proportion of patients who experienced improvement in visual deficits in the short period after surgery gradually increased, reaching 53% in the final interval. In other studies, the proportion of patients demonstrating improvement in visual deficits was higher, exceeding 70% and even reaching 95% in some cohorts [8,28,32]. The difference in outcomes may stem from the fact that we assessed short-term postoperative visual outcomes (up to 1 month) due to limited access to ophthalmological follow-up data. Therefore, short-term visual outcomes may underestimate the true functional benefit of surgery.

Three phases of recovery of visual function after decompression have been described, and visual improvement may continue for up to 3 years [32,33,34,35]. Some authors report that visual improvement—particularly in patients with severe preoperative impairment—may be minimal immediately after surgery and even within the first 3 months, reaching only 15.2% and 27.8%, respectively [34]. The short-term treatment effect may therefore be underestimated when compared with long-term analyses performed after 1 year. Many authors support the theory of time-dependent convalescence after tumor extirpation, consistent with current literature demonstrating significant improvements after 6 months but not after 3 months [32,34,35].

## 5. Complications

A high proportion of large tumors with cavernous sinus invasion in our cohort may also explain the increased rates of postoperative DI and CSF leakage compared with other studies. In our analysis, the rate of transient DI was high at 30.8%, whereas other authors have reported values as low as 9.1% [28]. Most studies do not report transient DI rates and instead focus on persistent DI. The incidence of persistent DI in our cohort was 4%, which was comparable to findings in other reports. Kim et al. reported a rate of 3%, whereas in the study by Qureshi et al., the initial incidence was 11%, decreasing to 5.8% in later periods [12,28].

In our study, CSF leakage occurred in 8 patients (6.5%), with 1, 2, 0, and 5 cases in the respective quartiles, showing a non-significant increasing trend. This may be related to the high proportion of large tumors with cavernous sinus invasion in our cohort, the non-significantly increasing number of Knosp grade 4 tumors in later quartiles, as well as a more aggressive attempt at achieving complete surgical resection alongside increasing surgeon experience. Similar observations were reported by Chi et al., who also found a higher postoperative CSF leakage rate in the late group [17]. In contrast, Younus et al. described a declining trend in CSF leakage, from 3% in the earliest quartile to 0.7% in the last; however, that study included a substantially lower proportion of Knosp grade 3 and 4 tumors (up to 29% in the third quartile), and the mean maximum tumor diameter was 23.3 mm [8]. In our cohort, the proportion of Knosp grade 3 and 4 tumors exceeded 50% in each quartile, and the mean maximum tumor diameter reached 29.4 mm. Interpretation of postoperative CSF leak trends should be cautious due to missing intraoperative leak data.

The surgical approach, instrumentation and reconstruction strategy remained consistent throughout the study period. No major technical modifications were introduced; therefore, observed changes in outcomes are primarily attributed to increasing surgical experience rather than technique evolution.

### Tumor-Related Factors Influencing the Assessment of the Learning Curve

When evaluating the learning curve in endoscopic endonasal surgery for pituitary tumors, several tumor-related factors can significantly confound the interpretation of surgical outcomes. Tumor size and cavernous sinus invasion are the most important variables, as larger adenomas and giant lesions are technically more demanding, often requiring extended exposure, more complex dissection, and increasing the risk of neurovascular injury. Consequently, these cases are associated with longer operative times, a higher risk of intraoperative CSF leakage, and potentially lower rates of gross total resection. If the surgeon’s case mix evolves over time—from predominantly smaller, less complex microadenomas in the early phase to larger and more invasive lesions later—this may obscure the apparent improvement in surgical performance.

Another important factor affecting the assessment of the learning curve is the number of cases performed per year. A low annual surgical volume may hinder the acquisition and consolidation of new skills, thereby prolonging the learning phase. Conversely, centers or surgeons with a higher case load tend to achieve proficiency more rapidly due to continuous exposure and experience. Therefore, when evaluating learning curves across studies or surgical teams, the annual number of procedures should be carefully considered as a potential bias influencing outcomes.

Without adjusting for these tumor-specific characteristics, the observed learning curve may not accurately reflect the surgeon’s true progression in skill, but rather differences in case complexity over time. Moreover, failure to adjust for tumor complexity may falsely suggest stagnation in surgical performance despite increasing technical proficiency.

In conclusion, relatively few studies have properly evaluated the learning curve in this field. Moreover, there is considerable variability in the methods used to report outcomes, which makes comparison between studies difficult. Given the presence of multiple confounding factors that may influence the assessment of the learning process, further research is needed to establish standardized evaluation methods. Such efforts are essential to better understand the dynamics of surgical skill acquisition and to adequately prepare neurosurgeons for these technically demanding procedures.

## 6. Study Limitations

This study has several limitations. Its retrospective design and single-center setting may limit generalizability. Although case complexity increased over time, the learning curve was analyzed in chronological order rather than by tumor characteristics, which could partially confound the interpretation of operative time reductions. Additionally, the small number of functioning adenomas limits the statistical power of endocrine outcome comparisons. Although this study includes a relatively large single-surgeon series, the sample size may still be insufficient to detect subtle improvements in resection rates after stratification by tumor invasiveness.

Another important limitation concerns the assessment of visual outcomes. In the majority of patients, visual function was evaluated only in the short-term postoperative period, while long-term ophthalmological follow-up was available in a limited subset of cases. Given that visual recovery after optic chiasm decompression may continue for several months or even years, the short-term assessment used in this study may underestimate the true extent of postoperative visual improvement.

Additionally, intraoperative CSF leak was not systematically recorded and therefore could not be analyzed. This limits the ability to correlate intraoperative CSF leak with postoperative leakage rates and to fully assess the evolution of skull base reconstruction techniques over time.

However, the homogeneous operative technique, consistent data collection, and relatively large sample size strengthen the validity of the findings.

## 7. Conclusions

1. A learning curve exists in endoscopic pituitary surgery, as demonstrated by a statistically significant reduction in operative time—also for FPA—as well as in the rate of de novo pituitary insufficiency.
2. The proportion of patients showing postoperative visual improvement and recovery from cranial nerve palsy gradually increased over time, although this trend did not reach statistical significance.
3. After approximately 50 endoscopic transsphenoidal surgeries, a surgeon may be considered well-trained or proficient, and after around 90 cases, experienced.
4. Tumor diameter, Knosp grade, and sphenoid sinus invasion were significantly associated with a lower rate of gross total resection.
5. Tumor size and cavernous sinus invasion appear to be factors that significantly confound the interpretation of surgical outcomes, such as the rate of GTR, postoperative CSF leakage, and DI. Learning curve assessment without adjustment for tumor invasiveness may lead to misleading conclusions.
6. The absence of an increasing GTR rate over time does not imply a lack of surgical progression but rather reflects a shift toward more complex cases. Operative efficiency and functional outcomes improved despite stable GTR rates, suggesting maturation of surgical skill.
7. Interpretation of visual and CSF-related outcomes should take into account the predominantly short-term visual assessment and the lack of systematic intraoperative CSF leak documentation. Future studies incorporating standardized long-term visual follow-up and systematic recording of intraoperative CSF leakage are needed to further refine outcome assessment in learning curve analyses
8. Future studies with larger cohorts and complexity-adjusted analyses are warranted to better elucidate the effect of surgical experience on resection rates.

## Figures and Tables

**Table 1:** Baseline demographic and clinical characteristics of the study cohort. Data are presented as the number of patients (percentage) or mean ± standard deviation (SD), unless otherwise indicated.

Variable	*n* (%) or Mean ± SD
Sex	
Female	54 (43.9%)
Male	69 (56.1%)
Mean age (years)	61.3 ± 13.9
Quartiles	
Q1	31 (25.2%)
Q2	28 (22.8%)
Q3	32 (26.0%)
Q4	32 (26.0%)
Procedure	
First surgery	114 (92.7%)
Recurrent surgery	9 (7.3%)
Patients with prior radiotherapy to the pituitary region	2 (1.6%)
Diabetes mellitus	26 (21.1%)
Active smoking	39 (31.7%)
Most common symptoms at diagnosis	
Subjective visual deterioration	64 (52.0%)
Headache	44 (35.8%)
Preoperative CSF leak	1 (0.8%)
Follow-up period, months	43.8 ± 27.6

**Table 2:** Tumor characteristics and surgical outcomes across quartiles of surgical experience.

Variable	Quartile 1 *n* (%)	Quartile 2 *n* (%)	Quartile 3 *n* (%)	Quartile 4 *n* (%)	*p* Value
Maximum tumor dimension (Mean ± SD)	29.0 ± 1.93	30.6 ± 2.0	28.3 ± 1.9	29.9 ± 1.9	0.843
Invasion of the sphenoid sinus	12 (44.4%)	11 (40.7%)	15 (46.9%)	15 (48.4%)	0.943
Knosp grade 0 *	1 (3.2%)	0	0	2 (6.3%)	0.580
Knosp grade 1 *	3 (9.7%)	7 (25.0%)	4 (12.5%)	0	0.053
Knosp grade 2 *	7 (22.6%)	3 (10.7%)	10 (31.3%)	9 (28.1%)	0.480
Knosp grade 3 *	9 (29.0%)	11 (39.3%)	9 (28.1%)	9 (28.1%)	0.960
Knosp grade 4 *	7 (22.6%)	5 (17.9%)	9 (28.1%)	11 (34.4%)	0.670
Non-functioning adenoma	25 (80.6%)	20 (71.4%)	23 (71.9%)	21 (65.6%)	0.614
Prolactinoma **	6 (19.4%)	5 (17.9%)	4 (12.5%)	6 (18.8%)	0.882
ACTH-secreting adenoma	0	0	0	1 (3.1%)	0.413
GH-secreting adenoma **	0	3 (10.7%)	5 (15.6%)	5 (15.6%)	0.145
Tumor recurrence	0	2 (7.1%)	0	1 (3.1%)	0.242
Gross total resection overall	13 (48.1%)	10 (38.5%)	17 (53.1%)	16 (51.6%)	0.720
Gross total resection for Knosp 0–2	9 (81.8%)	6 (60%)	11 (78.6%)	9 (81.8%)	0.690
Gross total resection for Knosp 3–4	4 (25%)	4 (25%)	6 (33.3%)	7 (35%)	0.860
Duration of surgery (min)	160.8 ± 54.5	109.3 ± 27.1	128.6 ± 42.8	109.7 ± 24.1	-
Length of hospital stay (days)	6.1 ± 3.0	6.0 ± 2.1	9.5 ± 5.0	8.1 ± 5.5	-

* Knosp grade data was available for 116 cases. ** One patient had an adenoma which was secreting both prolactin and growth hormone.

**Table 3:** Endocrinological, ophthalmological and cranial nerves outcomes across quartiles.

Variable	Q1 *n* (%)	Q2 *n* (%)	Q3 *n* (%)	Q4 *n* (%)	*p* Value
Preoperative pituitary insufficiency	7 (22.6%)	6 (21.4%)	13 (40.6%)	10 (31.2%)	0.314
New or worsened pituitary insufficiency	14 (45.2%)	19 (67.9%)	5 (15.6%)	8 (25.0%)	<0.001
Pituitary insufficiency improvement postoperatively	3 (9.7%)	3 (10.7%)	9 (28.1%)	5 (15.6%)	0.176
Secreting hormone reduction or normalization postoperatively	6 (100.0%)	8 (100.0%)	6 (66.7%)	6 (50.0%)	0.369
Preoperative visual deficits	21 (61.5%)	18 (62.9%)	19 (56.7%)	15 (46.9%)	0.563
Postoperative visual deficits	9 (34.6%)	13 (48.1%)	11 (36.7%)	7 (21.9%)	0.216
New postoperative vision disturbance (vision worsening)	0	0	0	1 (3.1%)	0.441
Preoperative cavernous sinus CN palsy	6 (15.4%)	1 (3.7%)	5 (13.3%)	3 (9.4%)	0.437
Postoperative cavernous sinus CN palsy	6 (15.4%)	0	2 (6.7%)	1 (3.1%)	0.160
Cavernous sinus CN palsy worsening	0	0	0	1 (3.1%)	0.441
Postoperative CSF leak	1 (3.2%)	2 (7.1%)	0	5 (15.6%)	0.067
Postoperative temporary DI	6 (19.4%)	11 (39.3%)	10 (31.2%)	11 (34.4%)	0.385
Postoperative persistent DI	2 (6.5%)	2 (7.1%)	1 (3.1%)	0 (0.0%)	0.464

## Data Availability

Data could be available upon request.

## References

[1] Liu J.K., Cohen-Gadol A.A., Laws E.R., Cole C.D., Kan P., Couldwell W.T. (2005). Harvey Cushing and Oskar Hirsch: Early forefathers of modern transsphenoidal surgery. J. Neurosurg..

[2] Eseonu C.I., ReFaey K., Rincon-Torroella J., Garcia O., Wand G.S., Salvatori R., Quinones-Hinojosa A. (2017). Endoscopic Versus Microscopic Transsphenoidal Approach for Pituitary Adenomas: Comparison of Outcomes During the Transition of Methods of a Single Surgeon. World Neurosurg..

[3] Doglietto F., Prevedello D.M., Jane J.A., Han J., Laws E.R. (2005). Brief history of endoscopic transsphenoidal surgery--from Philipp Bozzini to the First World Congress of Endoscopic Skull Base Surgery. Neurosurg. Focus.

[4] Jankowski R., Auque J., Simon C., Marchal J.C., Hepner H., Wayoff M. (1992). Endoscopic pituitary tumor surgery. Laryngoscope.

[5] Rotenberg B., Tam S., Ryu W.H., Duggal N. (2010). Microscopic versus endoscopic pituitary surgery: A systematic review. Laryngoscope.

[6] Al-Dardery N.M., Khaity A., Soliman Y., Ali M.O.M., Zedan E.M., Muyasarah K., Elfakhrany M.D. (2025). Safety and efficacy of endoscopic vs. microscopic approaches in pituitary adenoma surgery: A systematic review and meta-analysis. Neurosurg. Rev..

[7] Candy N.G., Ovenden C., Jukes A.K., Wormald P.J., Psaltis A.J. (2023). The learning curve for endoscopic endonasal pituitary surgery: A systematic review. Neurosurg. Rev..

[8] Younus I., Gerges M.M., Uribe-Cardenas R., Morgenstern P., Kacker A., Tabaee A., Anand V.K., Schwartz T.H. (2020). The slope of the learning curve in 600 consecutive endoscopic transsphenoidal pituitary surgeries. Acta Neurochir..

[9] Younus I., Gerges M.M., Uribe-Cardenas R., Morgenstern P.F., Eljalby M., Tabaee A., Greenfield J.P., Kacker A., Anand V.K., Schwartz T.H. (2020). How long is the tail end of the learning curve? Results from 1000 consecutive endoscopic endonasal skull base cases following the initial 200 cases. J. Neurosurg..

[10] Huang J., Hong X., Cai Z., Lv Q., Jiang Y., Dai W., Hu G., Yan Y., Chen J., Ding X. (2023). The learning curve of endoscopic endonasal transsphenoidal surgery for pituitary adenomas with different surgical complexity. Front. Surg..

[11] Leach P., Abou-Zeid A.H., Kearney T., Davis J., Trainer P.J., Gnanalingham K.K. (2010). Endoscopic transsphenoidal pituitary surgery: Evidence of an operative learning curve. Neurosurgery.

[12] Qureshi T., Chaus F., Fogg L., Dasgupta M., Straus D., Byrne R.W. (2016). Learning curve for the transsphenoidal endoscopic endonasal approach to pituitary tumors. Br. J. Neurosurg..

[13] Eseonu C.I., ReFaey K., Pamias-Portalatin E., Asensio J., Garcia O., Boahene K.D., Quiñones-Hinojosa A. (2018). Three-Hand Endoscopic Endonasal Transsphenoidal Surgery: Experience with an Anatomy-Preserving Mononostril Approach Technique. Oper. Neurosurg..

[14] Lofrese G., Vigo V., Rigante M., Grieco D.L., Maresca M., Anile C., Mangiola A., De Bonis P. (2018). Learning curve of endoscopic pituitary surgery: Experience of a neurosurgery/ENT collaboration. J. Clin. Neurosci..

[15] Smith S.J., Eralil G., Woon K., Sama A., Dow G., Robertson I. (2010). Light at the end of the tunnel: The learning curve associated with endoscopic transsphenoidal skull base surgery. Skull Base.

[16] Kassam A., Snyderman C.H., Carrau R.L., Gardner P., Mintz A. (2005). Endoneurosurgical hemostasis techniques: Lessons learned from 400 cases. Neurosurg. Focus.

[17] Chi F., Wang Y., Lin Y., Ge J., Qiu Y., Guo L. (2013). A learning curve of endoscopic transsphenoidal surgery for pituitary adenoma. J. Craniofac. Surg..

[18] O’Malley B.W., Grady M.S., Gabel B.C., Cohen M.A., Heuer G.G., Pisapia J., Bohman L.E., Leibowitz J.M. (2008). Comparison of endoscopic and microscopic removal of pituitary adenomas: Single-surgeon experience and the learning curve. Neurosurg. Focus.

[19] Shikary T., Andaluz N., Meinzen-Derr J., Edwards C., Theodosopoulos P., Zimmer L.A. (2017). Operative Learning Curve After Transition to Endoscopic Transsphenoidal Pituitary Surgery. World Neurosurg..

[20] Wang F., Zhou T., Wei S., Meng X., Zhang J., Hou Y., Sun G. (2015). Endoscopic endonasal transsphenoidal surgery of 1166 pituitary adenomas. Surg. Endosc..

[21] Dehdashti A.R., Ganna A., Karabatsou K., Gentili F. (2008). Pure endoscopic endonasal approach for pituitaryadenomas: Early surgical results in 200 patientsand comparison with previous microsurgical series. Neurosurgery.

[22] Gondim J.A., Schops M., de Almeida J.P., de Albuquerque L.A., Gomes E., Ferraz T., Barroso F.A. (2010). Endoscopic endonasal transsphenoidal surgery: Surgical results of 228 pituitary adenomas treated in a pituitary center. Pituitary.

[23] Cappabianca P., Cavallo L.M., de Divitiis E. (2004). Endoscopic endonasal transsphenoidal surgery. Neurosurgery.

[24] Koc K., Anik I., Ozdamar D., Cabuk B., Keskin G., Ceylan S. (2006). The learning curve in endoscopic pituitary surgery and our experience. Neurosurg. Rev..

[25] Boetto J., Joitescu I., Raingeard I., Ng S., Le Corre M., Lonjon N., Crampette L., Favier V. (2022). Endoscopic transsphenoidal surgery for non-functioning pituitary adenoma: Learning curve and surgical results in a prospective series during initial experience. Front. Surg..

[26] Bokhari A.R., Davies M.A., Diamond T. (2013). Endoscopic transsphenoidal pituitary surgery: A single surgeon experience and the learning curve. Br. J. Neurosurg..

[27] Shou X., Shen M., Zhang Q., Zhang Y., He W., Ma Z., Zhao Y., Li S., Wang Y. (2016). Endoscopic endonasal pituitary adenomas surgery: The surgical experience of 178 consecutive patients and learning curve of two neurosurgeons. BMC Neurol..

[28] Kim J.H., Lee J.H., Lee J.H., Hong A.R., Kim Y.J., Kim Y.H. (2018). Endoscopic Transsphenoidal Surgery Outcomes in 331 Nonfunctioning Pituitary Adenoma Cases After a Single Surgeon Learning Curve. World Neurosurg..

[29] Tabaee A., Anand V.K., Barrón Y., Hiltzik D.H., Brown S.M., Kacker A., Mazumdar M., Schwartz T.H. (2009). Predictors of short term outcomes following endoscopic pituitary surgery. Clin. Neurol. Neurosurg..

[30] Freda P.U., Beckers A.M., Katznelson L., Molitch M.E., Montori V.M., Post K.D., Vance M.L. (2011). Endocrine Society Pituitary incidentaloma: An endocrine society clinical practice guideline. J. Clin. Endocrinol. Metab..

[31] Lissett C.A., Peacey S.R., Laing I., Tetlow L., Davis J.R., Shalet S.M. (1998). The outcome of surgery for acromegaly: The need for a specialist pituitary surgeon for all types of growth hormone (GH) secreting adenoma. Clin. Endocrinol..

[32] Gnanalingham K.K., Bhattacharjee S., Pennington R., Ng J., Mendoza N. (2005). The time course of visual field recovery following transphenoidal surgery for pituitary adenomas: Predictive factors for a good outcome. J. Neurol. Neurosurg. Psychiatry.

[33] Kerrison J.B., Lynn M.J., Baer C.A., Newman S.A., Biousse V., Newman N.J. (2000). Stages of improvement in visual fields after pituitary tumor resection. Am. J. Ophthalmol..

[34] Butenschoen V.M., Schwendinger N., von Werder A., Bette S., Wienke M., Meyer B., Gempt J. (2021). Visual acuity and its postoperative outcome after transsphenoidal adenoma resection. Neurosurg. Rev..

[35] Moon C.H., Hwang S.C., Ohn Y.H., Park T.K. (2011). The time course of visual field recovery and changes of retinal ganglion cells after optic chiasmal decompression. Investig. Ophthalmol. Vis. Sci..

